# Computational prediction of multidisciplinary team decision-making for adjuvant breast cancer drug therapies: a machine learning approach

**DOI:** 10.1186/s12885-016-2972-z

**Published:** 2016-12-01

**Authors:** Frank P. Y. Lin, Adrian Pokorny, Christina Teng, Rachel Dear, Richard J. Epstein

**Affiliations:** 1 Department of Oncology, St Vincent’s Hospital, The Kinghorn Cancer Centre, 370 Victoria St, Darlinghurst, Sydney, Australia; 2 Garvan Institute of Medical Research, Sydney, Australia; 3 The University of New South Wales, Sydney, NSW Australia; 4 The University of Sydney, Sydney, NSW Australia

**Keywords:** Breast cancer, Cytotoxic drug therapy, Decision analysis, Machine learning, Clinical decision support system

## Abstract

**Background:**

Multidisciplinary team (MDT) meetings are used to optimise expert decision-making about treatment options, but such expertise is not digitally transferable between centres. To help standardise medical decision-making, we developed a machine learning model designed to predict MDT decisions about adjuvant breast cancer treatments.

**Methods:**

We analysed MDT decisions regarding adjuvant systemic therapy for 1065 breast cancer cases over eight years. Machine learning classifiers with and without bootstrap aggregation were correlated with MDT decisions (recommended, not recommended, or discussable) regarding adjuvant cytotoxic, endocrine and biologic/targeted therapies, then tested for predictability using stratified ten-fold cross-validations. The predictions so derived were duly compared with those based on published (ESMO and NCCN) cancer guidelines.

**Results:**

Machine learning more accurately predicted adjuvant chemotherapy MDT decisions than did simple application of guidelines. No differences were found between MDT- *vs.* ESMO/NCCN- based decisions to prescribe either adjuvant endocrine (97%, *p* = 0.44/0.74) or biologic/targeted therapies (98%, *p* = 0.82/0.59). In contrast, significant discrepancies were evident between MDT- and guideline-based decisions to prescribe chemotherapy (87%, *p* < 0.01, representing 43% and 53% variations from ESMO/NCCN guidelines, respectively). Using ten-fold cross-validation, the best classifiers achieved areas under the receiver operating characteristic curve (AUC) of 0.940 for chemotherapy (95% C.I., 0.922—0.958), 0.899 for the endocrine therapy (95% C.I., 0.880—0.918), and 0.977 for trastuzumab therapy (95% C.I., 0.955—0.999) respectively. Overall, bootstrap aggregated classifiers performed better among all evaluated machine learning models.

**Conclusions:**

A machine learning approach based on clinicopathologic characteristics can predict MDT decisions about adjuvant breast cancer drug therapies. The discrepancy between MDT- and guideline-based decisions regarding adjuvant chemotherapy implies that certain non-clincopathologic criteria, such as patient preference and resource availability, are factored into clinical decision-making by local experts but not captured by guidelines.

**Electronic supplementary material:**

The online version of this article (doi:10.1186/s12885-016-2972-z) contains supplementary material, which is available to authorized users.

## Background

Decision-making in modern cancer treatment is a complex process that requires coordinated expertise from surgeons, oncologists, radiologists, pathologists, and allied health professionals. Multidisciplinary team (MDT, ‘tumour board’) meetings are now routinely held to integrate these diverse management inputs, and have led to significant improvements in evidence-based decision-making and care quality [1, 2]. Patient-related benefits from MDTs include improved survival, fewer invasive interventions, greater medical staff efficiency, and enhanced quality of life [3, 4].

MDTs augment clinical decision-making by reconciling multiple viewpoints of an individual patient’s problem [1]. With respect to implementation, there are two main obstacles that limit the value of MDT decision-making. First, the specialist expertise from a single institution cannot be readily contributed to other institutions servicing different patient casemixes; the adoption of practice guidelines aims to address this issue, but such broad-brush approaches are problematic to apply to unique or complex cases. Consequently, while guidelines may aid decision making, adherence to the recommendations is often suboptimal [5, 6]. In early breast cancer, co-morbidities, behavioural, and resource barriers limit applicability to individual patients, leading to deviations [6–8]; a substantial discrepancy between the major guidelines also exists [9]. Second, the quality of MDT decision-making is not readily evaluable or capable of standardisation, though methodologies have been developed to this end [3, 10].

One strategy to address the foregoing problems is to use data captured from routine MDTs to derive models that systematically predict the decisions made therein. If reliable data-driven models could be developed, this would facilitate dissemination of expertise, provide automatic decision support, and permit data audit in a health service context. Here we have hypothesised that the decisions made in a cancer MDT may be predicted by supervised machine learning methods. To test this hypothesis, we have sought to develop models that predict MDT recommendations about adjuvant systemic treatments in early breast cancer.

## Methods

### Study population

We conducted a single-centre study at a tertiary cancer referral centre in Sydney, Australia. Clinicopathologic data from consecutive cases presented to a weekly breast cancer MDT from January 2007 through March 2015 were screened. The MDT discussion process took place by first examining the relevant clinical, histopathology, imaging, and surgical findings by a panel of experts (consists of surgeons, pathologists, radiologists, oncologists, and allied health professionals) followed by an open discussion to reach the final recommendations about further investigations, additional surgery, or adjuvant treatments. Patients with a new diagnosis of early breast cancer who underwent a curative resection (wide local excision, partial mastectomy, or mastectomy) were including in the analysis. Cases excluded from the analysis included those presented prior to the definitive surgical resection, with metastatic disease at the time of presentation, and those limited to benign or non-invasive histology type (for example, ductal carcinoma *in situ*, DCIS, or lobular carcinoma *in situ*, LCIS). A case was also excluded if none of the oestrogen receptor (ER), progesterone receptor (PR), and human epithelial growth factor receptor 2 (HER2) statuses was recorded. Cases without at least one of the three adjuvant systemic therapy decisions (i.e. chemotherapy, endocrine therapy, or trastuzumab - biologic/targeted - therapy) were also excluded from the analysis.

### Independent variables

Variables included in the analysis are enumerated in Additional file 1: Table S1. These comprise the year the MDT was held; demographics of the patient; menopausal status; prior treatment; nodal status (both sentinel and/or axillary lymph nodes status, if conducted); cell types; histological grade; size of primary tumour; presence of lymphovascular or perineural invasions; margin status from the surgery; ER/PR/HER2 status; Cytokeratin 5/6; Ki-67; whether a second primary was present; the presence of DCIS and LCIS; and tumour size. Luminal A-like histology was defined as ER+, Ki-67 ≤ 14%, HER2-negative), whereas luminal B type histology was defined as ER+, Ki-67 ≥ 15%, *or* ER+, HER2 2+ on IHC, FISH non-amplified.

### Decision outcome characterisation

Decision outcomes from the MDT were discretised into three categories: (1) *recommended*, where a given treatment modality is recommended by the MDT, (2) *not recommended*, where the MDT consensus is against the administration of the treatment modality, or (3) *for discussion*, where the patient may or may not be considered for the treatment modality, depending in part on their reaction to a full discussion of possible risks and benefits of taking either a pro-active or observation-only treatment approach. To capture both potential extremes of recommendation, the three-way decision was further dichotomised into two binary strategies, viz., the *aggressive strategy* (in which all “for discussion” cases are assumed to be ultimately “recommended”) vs. the *conservative strategy* (in which all “for discussion” cases are assumed to be ultimately “not recommended”).

### Predictive modelling with supervised machine learning algorithms

Supervised machine learning encompasses a wide range of computational methods that use historical data to train models for predicting the outcomes of new cases. To determine which model type best predicted MDT decisions, we systematically examined 10 supervised machine learning classifiers from distinct classes include naïve Bayesian classifier, support vector machines with polynomial and radial basis function kernels, multivariate logistic regression, nearest neighbours, ripple down rules, J48 and alternating decision trees. Bootstrap aggregation was applied (using 10 bootstrap steps) on eight of the ten models. The parameters used for model training are listed in Additional file 1: Table S2. The out-of-sample classifier performance was assessed by area under the receiver operating characteristic curve (AUC) estimated by stratified ten-fold cross-validation. The confidence intervals of AUC were estimated by using the Hanley-McNeil method [11].

### Comparison with major practice guidelines

For each case, final MDT decisions of all modalities were compared against the corresponding recommendations by the algorithms specified in the European Society for Medical Oncology (ESMO) and National Comprehensive Cancer Networks (NCCN) guidelines published in the immediate preceding year(s) using the same clinicopathological variables [12–16]. A decision branch was treated as “for discussion” if a recommendation was labelled “consider” or “± modality” (for example, ± chemotherapy) as denoted in the NCCN guidelines. The proportions of cases where the MDT recommendations agree with the guideline were recorded. Another view of the concordance of decisions involved measurement of how accurate the guidelines are used to “predict” MDT decisions on a case-by-case basis.

**Fig. 1:**
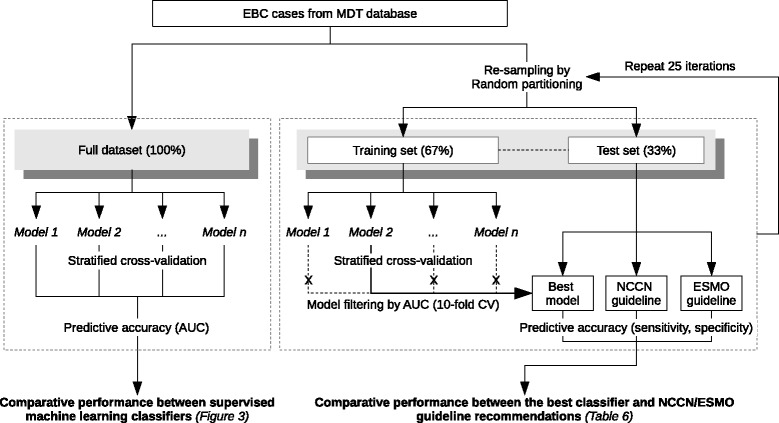
The analytic approach for comparing performance between machine learning classifiers and NCCN/ESMO guidelines

For the dichotomised groupings (i.e., the aggressive and conservative approaches), we also evaluated the sensitivity and specificity of each guideline for predicting against the corresponding MDT outcome. Both statistics were compared with the corresponding best classifier for each modality-strategy combination. A “wrapper-based” approach was used for comparing the performance between the best classifier and the two guidelines (Fig. 1): (1) Two-third of data (training and validation set) was used for selecting f the best model (i.e. the model with best mean AUC in stratified ten-fold validation), (2) the remaining one-third of data (test set) was used to estimate the sensitivity and specificity of method for classifying MDT decision about a treatment modality, and (3) the process is repeated twenty-five times and the mean measures were obtained.

### Statistical and ethics considerations

This study conformed to local ethical guidelines, and was approved by the Human Research Ethics Committee at the primary study institution. Waikato Environment for Knowledge Analysis (WEKA) version 3.6.6 was used for classifier training and evaluation [17]. The R statistical environment version 3.2.0 was used for statistical analysis. Custom PERL scripts were used for data cleaning, experimental pipeline, and aggregated analysis.

## Results

**Fig. 2:**
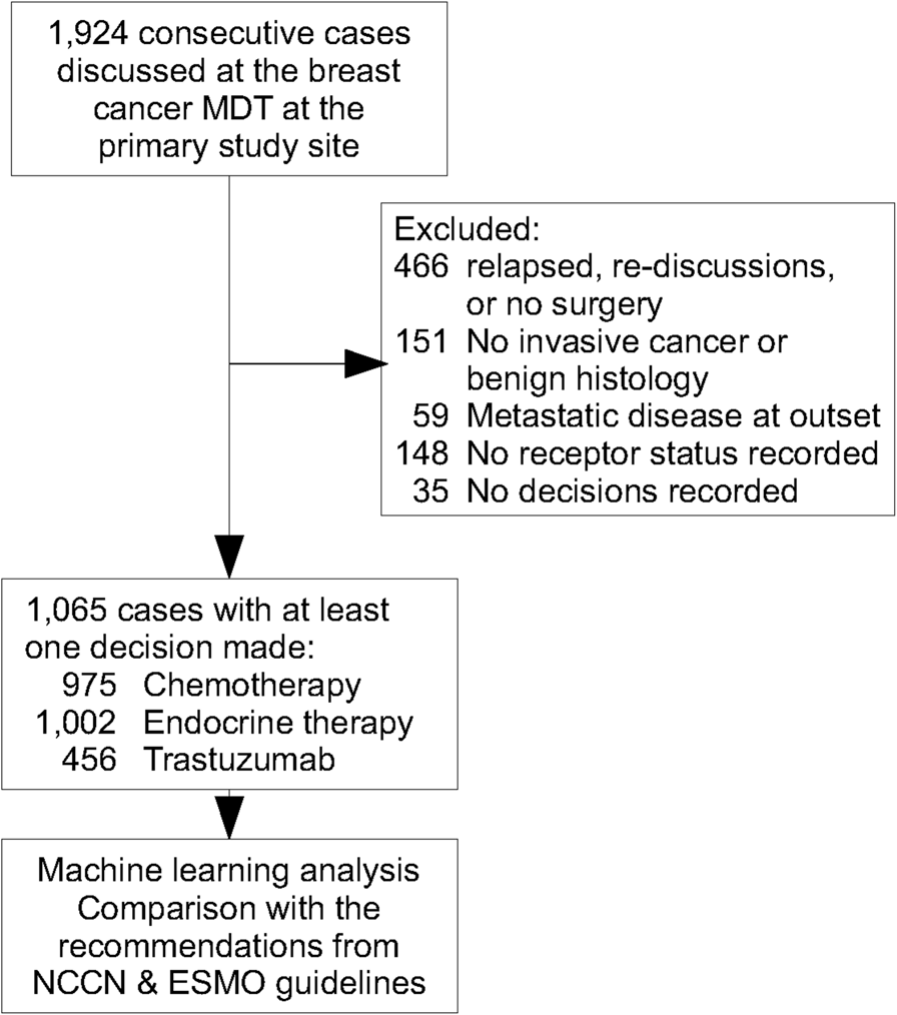
Flow diagram of the early breast cancer cases screened and included in the data analysis

From 1,924 cases screened, 1,065 cases were eligible for inclusion in the predictive analysis (Fig. 2). Patient characteristics are shown in Table 1. Most cases were female (1,053 cases, 99%). Histological subtypes of breast cancer included 633 patients with luminal-A-like tumour (59%), 294 patient with luminal-B-like tumour (28%), 95 were basal/triple-negative type (9%), and 43 with solely HER2 over-expressed (4%). Adjuvant chemotherapy was recommended in 342 (35%) of cases, whereas endocrine therapy and trastuzumab therapy were recommended in 794 (79%) and 86 (19%) of cases, respectively (Table 2).

**Table 1:** Baseline characteristics of early breast cancer cases discussed at the index MDT

Patient characteristics		Group	N	(%)
Demographics	Age group (years)	<45	141	(13)
		45-55	242	(23)
		56-69	393	(37)
		≥70	266	(25)
Surgery type	Primary tumour	WLE or partial mastectomy	608	(57)
		Total mastectomy	452	(42)
	Lymph node	Sentinel node biopsy only	703	(66)
		Axillary lymph node dissection	255	(24)
Nodal status	Sentinel lymph node	Involved	269	(25)
		Not involved	590	(55)
	Axillary lymph nodes involved	No	625	(59)
		1-3	240	(23)
		≥4	108	(11)
	Extranodal spread	Present	140	(13)
Histopathology	Cell type	Invasive ductal carcinoma	818	(77)
		Invasive lobular carcinoma	135	(13)
		Tubular carcinoma	27	(3)
		Mucinous carcinoma	12	(1)
		Medullary carcinoma	8	(0.8)
		Mixed type	8	(0.8)
		Basal type	7	(0.7)
		Metaplastic carcinoma	7	(0.7)
		Other malignant tumour	42	(4)
	Multifocal	Multifocal	83	(9)
		Satellite lesions	66	(6)
	Primary tumour size (cm)	≤0.5 (T1a)	42	(4)
		0.6-1.0 (T1b)	149	(14)
		1.1-2.0 (T1c)	423	(40)
		2.1-5.0 (T2)	347	(33)
		>5.0 (T3)	79	(7)
	Histological grade	Grade 1	172	(12)
		Grade 2	445	(42)
		Grade 3	418	(39)
	Lymphovascular invasion	Present	356	(33)
		Absent	308	(29)
	Perineural invasion	Present	40	(4)
		Absent	111	(10)
	Oestrogen receptor (ER) status	Positive	927	(87)
		Negative	135	(13)
	Progesterone receptor (PR) status	Positive	843	(79)
		Negative	210	(20)
	HER2 status^a^	Positive	128	(12)
		Negative	654	(61)
	Basal Type	Yes	31	(3)
	Cytokeratin 5/6	Positive	59	(6)
	Ki-67 (%)	<5%	94	(9)
		5-9%	130	(12)
		10-29%	231	(22)
		≥30%	153	(14)
Associated lesions	Second primary	Present	31	(3)
	Ductal carcinoma *in situ*	High Grade	335	(32)
		Intermediate Grade	196	(18)
		Low grade	43	(4)
		Extensive	346	(33)
		Focal	318	(30)
	Lobular carcinoma *in situ*	Focal	89	(8)
		Extensive	60	(6)
	Other benign lesion(s)	Present	95	(9)

*WLE* Wide local excision

NB: ^a^HER2 status as determined by *in situ* hybridisation

**Table 2:** Summary of systemic adjuvant treatment recommendations by modality and expertise

Adjuvant treatment				Recommendation					
Modality	Expertise	Recorded		Recommended		Not Recommended		For discussion	
		N	(%)	N	(%)	N	(%)	N	(%)
Chemotherapy	MDT	975	(92)	342	(35)	393	(40)	240	(25)
	ESMO	1065	(100)	321	(30)	701	(66)	43	(4)
	NCCN	1065	(100)	489	(46)	80	(8)	496	(47)
Endocrine therapy	MDT	1002	(94)	794	(79)	86	(9)	122	(2)
	ESMO	1065	(100)	927	(87)	138	(13)	0	(0)
	NCCN	1065	(100)	874	(82)	143	(13)	48	(5)
Trastuzumab	MDT	456	(43)	86	(19)	363	(80)	7	(2)
	ESMO	1065	(100)	142	(13)	923	(87)	0	(0)
	NCCN	1065	(100)	125	(12)	929	(87)	11	(1)

*MDT* multidisciplinary conference, *ESMO* European society for medical oncology, *NCCN* national comprehensive cancer network

**Fig. 3:**
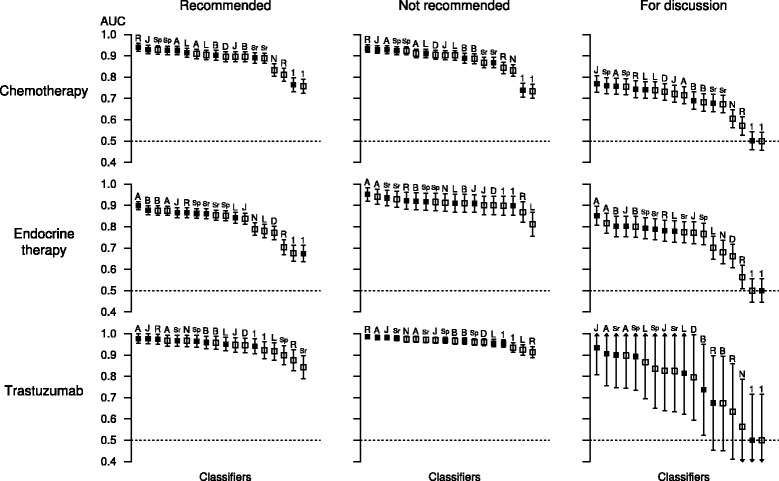
Performance of machine learning models for predicting the MDT decisions. Each point indicates the mean AUC (from ten cross-validation runs) of a classifier for correctly predicting the outcome of MDT recommendation. The error bars indicate the 95% confidence intervals estimated by the Hanley-McNeil method. The open square indicate the classifiers *without* bootstrap-aggregation, whereas the solid squares indicate the corresponding classifiers *with* bootstrap-aggregation. Legend: R: ripple down rule, *J* J48 classifier. A: multiclass alternating decision tree, *Sp* support vector machine (SVM) with polynomial kernel, *Sr* SVM with radial basis function kernel, *D* decision Table 1: OneR classifier, *B* naive Bayesian classifier, *N* nearest neighbour classifier, *L* Multivariate logistic regression

Bootstrap-aggregated (bagged) decision trees [multiclass alternating decision tree (ADTree) and J48 decision tree] proved superior to probabilistic models, support vector machines, and un-bagged models (Fig. 3). The best algorithm for predicting whether adjuvant chemotherapy should be recommended was bagged ripple-down rules (AUC 0.940, 95% CI: 0.922—0.958), whereas the bagged multiclass ADTree was the algorithm of choice for both endocrine therapy (AUC 0.899, 95% CI: 0.880 - 0.918) and trastuzumab (AUC 0.977, 95% CI: 0.955 - 0.999) respectively. The multivariate logistic regression performed on average of chemotherapy with an AUC of 0.904 (95% CI: 0.881 - 0.927), endocrine therapy (AUC 0.780, 0.749 - 0.811), trastuzumab (AUC 0.917, 0.876 - 0.958) respectively. A separate multivariate logistic regression analysis was performed to list the key clinicopathologic factors that contribute to the recommendation of adjuvant chemotherapy by the breast MDT (Table 3). Performance of classifiers for predicting all treatment-recommendation combinations is summarised in Fig. 3 and is further illustrated in detail in Additional file 1: Figures S1-S3. The predictive co-variates identified by supervised learning are listed in Additional file 1: Table S3.

**Table 3:** Multivariate logistic regression model showing the key clinicopathologic factors contributing to the MDT recommendation of chemotherapy in early breast cancer

Variable	OR	95% C.I.
Age (per year)	0.86	(0.83–0.89)
Number of involved axillary lymph nodes (per node)	1.42	(1.22–1.64)
Primary tumour size (per mm)	1.04	(1.02–1.06)
Histology - Grade 3	14.5	(2.59–81.5)
Oestrogen receptor (ER) - positive	0.12	(0.038–0.37)
HER2 (by *in situ* hybridisation assay) - positive	14.2	(5.37–37.6)
Ki-67 (%, per 1% increase)	1.02	(1.0–1.04)

A separate analysis using maximum-likelihood multivariate logistic regression of seven variable first identified by the *cfsSubset* feature selection algorithm using best-first search strategy [26]

*Abbreviation*: *OR* The odds ratio of adjuvant chemotherapy being recommended by the MDT. Results from this table were presented as a scientific poster at the Annual Scientific Meeting of the Medical Oncology Group Australia, Surfers Paradise, Australia, 2-5 August 2016 [27]

A similar trend of classifier performance was observed for prediction of MDT decisions recommending against the administration of a particular treatment modality (Fig. 2). The accuracy of models for predicting the “for discussion” group was inferior to the definitive binary decisions, reflecting predictably heterogeneous decisions in this group. The predictive performance of almost all classifiers differed from chance (AUC of 0.5) at the type I error rate at α = 0.01 (two-sided, after adjustment for multiple hypothesis testing) for the “recommended” and “not recommended” classes. The overall median rank of each algorithm is listed in Table 4.

**Table 4:** Relative performance of machine learning algorithm across all therapy-recommendation combinations

Algorithm	Median rank
Multiclass ADTree (Bagged)	2.0
J48 decision tree (Bagged)	3.0
Ripple down rules (Bagged)	3.0
Multiclass ADTree	5.0
SVM, polynomial kernel (Bagged)	6.0
SVM, radial basis function kernel (Bagged)	7.0
SVM, polynomial kernel	7.0
SVM, radial basis function kernel	9.0
Naive Bayes classifier (Bagged)	9.0
Logistic regression (Bagged)	10.0
Naive Bayes classifier	11.0
J48 decision tree	11.0
Decision table	12.0
Logistic regression	14.0
Nearest neighbour classifier	14.0
Ripple down rules	16.0
OneR (Bagged)	17.0
OneR	17.0

*Abbreviations*: *Bagged* bootstrap-aggregated, *ADTree* alternating decision tree, *SVM* support vector machine

We then compared the machine learning approach with two international guidelines on the use of adjuvant systemic treatment for early breast cancer. The proportion of agreement between the MDT decision and the ESMO/NCCN guidelines is detailed in Table 5. MDT decisions about adjuvant endocrine and trastuzumab therapies were in close agreement with guidelines (85 and 96% respectively). For chemotherapy decisions, however, significant discrepancies were apparent between MDT- and guideline-based decisions (57% and 47% for ESMO and NCCN recommendations respectively). Of note, poor agreement (30%) was also evident between the two chemotherapy guidelines themselves. This latter discrepancy appeared mainly attributable to two factors: (i) use of the 21-gene panel in the ER-positive, HER2-negative (Luminal-A like) subtype – recommended by NCCN but not ESMO, and (ii) different treatment thresholds for patients with ‘oligonodal’ (one to three involved nodes) disease. Even with dichotomised decisions (aggressive or conservative), the concordance of MDT-based vs. guideline-based decisions only reached ~75%. These data imply that factors other than specified clinicopathological classifiers govern expert MDT decisions about adjuvant chemotherapy, but not about hormone therapy or trastuzumab.

**Table 5:** Pairwise comparison of the recommendations from the index MDT versus ESMO and NCCN guidelines

Treatment Modality	Agreement between the expertise					
Strategy	MDT versus ESMO		MDT versus NCCN		ESMO versus NCCN	
	N	(%)	N	(%)	N	(%)
Chemotherapy						
Overall^a^	551/975	(57)	462/975	(47)	320/1065	(30)
Aggressive	628/975	(64)	616/975	(63)	416/1065	(39)
Conservative	729/975	(75)	731/975	(75)	721/1065	(68)
Endocrine therapy						
Overall^a^	853/1002	(85)	840/1002	(84)	976/1065	(92)
Aggressive	970/1002	(97)	953/1002	(95)	1024/1065	(96)
Conservative	858/1002	(86)	861/1002	(86)	976/1065	(92)
Trastuzumab						
Overall^a^	437/456	(96)	437/456	(96)	1047/1065	(98)
Aggressive	444/456	(97)	446/456	(98)	1058/1065	(99)
Conservative	437/456	(98)	440/456	(97)	1047/1065	(98)

*Abbreviations*: *MDT* multidisciplinary team meeting, *ESMO* European Society for Medical Oncology guideline, *NCCN* National Comprehensive Cancer Network guideline

^a^Overall – three-way grouping of “Recommended”, “For discussion”, “Not recommended”

We further compared the predictive power of the machine learning models and guidelines for predicting adjuvant therapy decisions. In general, the machine learning-based approach predicted MDT decisions better than either ESMO or NCCN guidelines. At the default classifiers threshold, the positive likelihood ratios (LR+) for the best classifiers were 8.8 for chemotherapy (95% C.I.: 4.6 – 16.9), 6.5 for endocrine therapy (95% C.I.: 3.17 – 13.5), and 77.9 for trastuzumab therapy (95% C.I.: 7.1 – 858) for the aggressive grouping. Machine learning methods were non-inferior to guidelines in all treatment modality-strategy combinations (Table 6). In the conservative analysis of endocrine and trastuzumab therapy, both ESMO and NCCN guidelines were concordant with MDT decisions, as demonstrated by the high sensitivities, suggesting a value for guidelines in excluding patients who do not need treatment. No differences in the predictive performance were observed between endocrine or trastuzumab therapy between the best classifier and either guideline.

**Table 6:** The sensitivity, specificity, and positive likelihood ratio of predicting the index MDT decisions using the best machine learning model versus using ESMO and NCCN guidelines

MDT recommendation		Accuracy of prediction by							
Modality	Recommended	Best ML model^a^		ESMO Guidelines			NCCN Guidelines		
Strategy	N (%)	Sens/Spec	LR+	Sens/Spec	LR+	P^b^	Sens/Spec	LR+	P^b^
Chemotherapy									
Aggressive	582 (60)	0.93/0.89	8.8	*0.55* ^*c*^ */0.78* ^*c*^	2.5	<0.01	0.97/*0.12* ^*c*^	1.1	<0.01
Conservative	342 (35)	0.86/0.95	16.7	*0.60* ^*c*^ */0.82* ^*c*^	3.3	<0.01	0.82/*0.71* ^*c*^	2.9	<0.01
Endocrine									
Aggressive	916 (91)	0.98/0.85	6.5	0.98/0.81	5.2	0.68	0.97/0.75	3.9	0.25
Conservative	794 (79)	0.97/0.65	2.8	0.99/*0.36* ^*c*^	1.5	0.30	0.96/0.50	1.9	0.37
Trastuzumab									
Aggressive	93 (20)	0.98/0.99	77.9	0.97/0.97	33.3	0.60	0.97/0.98	45.2	0.73
Conservative	86 (19)	0.95/0.99	122.9	0.97/0.96	24.0	0.24	0.92/0.98	37.7	0.28

The sensitivity (sens), specificity (spec), and the positive likelihood ratio (LR+) when using the best machine learning models or guideline to predict MDT recommendations

Note: ^a^The best models were ripple down rules for the chemotherapy decisions, polynomial SVM for the aggressive endocrine decisions, and ADTree for the remaining groups

^b^pairwise comparisons of likelihood ratios using two-sided *z*-test (i.ebest model vs. guideline)

^c^the best model performed better than the guideline

## Discussion

The central findings of this study are two-fold. First, a machine learning-based approach is useful for predicting MDT decisions about adjuvant drug therapies in early breast cancer patients; to the best of our knowledge, this is the first systematic analysis of predictive modelling of the MDT outcome in breast cancer. Second, unlike adjuvant hormone or trastuzumab MDT decisions, adjuvant chemotherapy MDT decisions differed significantly from guideline-based decisions, suggesting that additional non-clinicopathologic variables impact upon expert advice in the adjuvant chemotherapy context. These findings could reflect chemotherapy-specific decision variations due to divergences in patient preference, cultural or socioeconomic differences, and resource availability. Since machine learning remained predictive of MDT decisions, we speculate that future work may succeed in identifying these important missing data, and thus help to understand this discrepancy better.

For early breast cancer patients, oncologists and their professional colleagues must determine the most appropriate adjuvant therapy. A multidisciplinary approach is important in making decisions about adjuvant treatments after a surgical resection with curative intent; the goals of recurrence reduction (deferral, cure) must be carefully weighed against the toxicity, cost, inconvenience and other detriments to patient quality of life. Although MDT opinions on whether a patient should undergo toxic treatment can be contentious between experienced clinicians, the benefits of a multidisciplinary approach clearly reduce breast cancer-specific mortality [18].

The goal of our modelling differs from prognosis-based decision aids such as Adjuvant! and the PREDICT Tool [19, 20], where the primary goal of these tools is to estimate benefits for a given level of risk for recurrence and/or death. A practical objective of our study is therefore to assess the feasibility of predicting the actual MDT outcome, which captures the practical aspects other than solely the survival considerations of a patient.

We found that the machine learning models were high discriminative of the outcome variables, with the predictive accuracy consistently achieved at a clinically useful level. The internal validity was demonstrated by thorough cross-validation evaluations. Further studies at an external centre would clarify its clinical utility. We expect our analytic approach could also predict MDT recommendations for other treatment modalities such as surgery and radiotherapy, as well as assist in decision-making for patients suffering metastatic disease.

Our analysis is strengthened by a comprehensive survey of classifiers with distinct inference techniques; the comparative design has allowed determination of the best algorithm for each task. The alternating decision tree algorithm outperformed other classifiers for predicting MDT decisions about endocrine and trastuzumab therapies; on the other hand, the bootstrap-aggregated ripple-down-rules classifier was superior for predicting adjuvant chemotherapy decisions. We conclude from this that a tree-based approach resembles more closely how experts make actual decisions in a collaborative environment. Conversely, both generative and discriminative probabilistic methods (such as naïve Bayesian classifier and multivariate logistic regression) did not perform as well as tree-based classifiers; one explanation for this may be that these algorithms were compromised by strong co-linearity between certain variables. Aggressive feature selection may thus be required to optimise their performance.

For decisions about adjuvant chemotherapy, significant discrepancies were apparent between MDT decisions and the two international guidelines. Guideline-driven individualisation of treatments may thus prove challenging; factors such as treatment toxicity, performance status, quality of life, psychological well-being, and patient's perception of treatment efficacy can strongly influence the treatment decision [8, 21–23], but such nuances are poorly captured by practice guidelines. Consequently, while evidence-based guidelines are designed to suit the majority of patients, our study highlighted the importance of individualised, patient-centred assessments as per best MDT practice. Identification of putative underlying non-clinicopathologic variables through a machine learning approach could help to elucidate how clinicians arrive at MDT decisions about adjuvant chemotherapy for early breast cancer.

A potential use of our modelling approach is to allow estimation of decision consistency within a cancer MDT. Intuitively, the most accurate model also indicates how well an MDT outcome can be predicted using the same clinical and pathological characteristics. A comparative evaluation of multiple models hence provides an objective mean for which the auditing of decision quality can be conducted within and/or between cancer centres.

Several applications are made possible by the machine learning approach described here. First, the most predictive classifier(s) can be packaged into a site-specific decision support system to help real-time decision making in a MDT, which has the potential to enhance the decision making process by considering local resource constraints compared with using an external guideline. The use of a computerised decision support can also improve uptake of evidence-based care [24]. Second, a reliable model should enable transfer of knowledge to smaller or less experienced centres, for example, in remote or rural settings, thus permitting early triage or referral of complex cases. Third, the decision about individual cases can be compared across different centres, which would otherwise not be feasible to do.

It is important to acknowledge that our study has several limitations. First, our data did not fully record the sequencing of treatment modalities, investigations, or chemotherapy regimens, which would otherwise allow us to fine-tune the predicted recommendations. Second, final decisions after patient review by medical oncologists (i.e., as distinct from the “intention to treat” recommendations recorded in MDTs) were not always available to us; we expect that these final treatment outcomes are modified by additional elements of patient preference. Third, survival benefits were unable to be quantified from our non-randomised (retrospective) data, since early breast cancer patients have a relatively good prognosis; a very large sample size with lengthy follow up would be required to draw meaningful conclusions on survival benefit. Fourth, our data did not fully record all administrative confounders, such as absence of a specific expert(s) from the MDT, delays in assessment, or attendance of the meeting. It is known that the team, social, and information factors do influence decisions made in a MDT [25]. A prospective study aiming to address these issues would thus be important to support solid models in the future. Finally, the present study represent only the expertise from a single cancer centre and hence may not reflect clinical practice elsewhere, though supervised learning approach can be readily extended to aggregate expertise from multiple centres. Despite the limitations, the demonstrated predictive accuracy of our study supports the future research studies of the machine learning model in a clinical setting.

## Conclusions

In summary, the present study demonstrates that the machine learning approach is indeed a useful method for predicting MDT decisions about adjuvant systemic therapy in early breast cancer, with better accuracy than using accepted therapeutic guidelines. This approach has the potential to provide direct decision support and facilitate transfer of local expertise to more remote centres, and hence to improve patient quality of care and clinical cancer outcomes.

## Additional file

Additional file 1:
**Table S1.** List of clinicopathologic and outcome variables used in this analysis. **Table S2.** Parameters and commands used for training of the supervised learning classifiers. **Table S3.** List of covariates used by the trained machine learning models. **Figure S1.** Predictions of MDT recommendations by machine learning algorithms about adjuvant chemotherapy for each case. **Figure S2.** Predictions of MDT recommendations by machine learning algorithms about adjuvant endocrine therapy for each case. **Figure S3.** Predictions of MDT recommendations by machine learning algorithms about adjuvant trastuzumab therapy for each case. (PDF 864 kb)

## Abbreviations

ADTree: Alternating decision tree
AUC: Area under the receiver operating characteristic (ROC) curve
DCIS: Ductal carcinoma *in situ*
ER: Oestrogen receptor
ESMO: European Society for Medical Oncology
HER2: Human epithelial growth factor receptor 2
LCIS: Lobular carcinoma *in situ*
MDT: Multidisciplinary team
NCCN: National Comprehensive Cancer Network
PR: Progesterone receptor
WEKA: Waikato Environment for Knowledge Analysis

## References

[1] Taylor C, Munro AJ, Glynne-Jones R, Griffith C, Trevatt P, Richards M, Ramirez AJ (2010). Multidisciplinary team working in cancer: what is the evidence?. BMJ.

[2] Saini KS, Taylor C, Ramirez AJ, Palmieri C, Gunnarsson U, Schmoll HJ, Dolci SM, Ghenne C, Metzger-Filho O, Skrzypski M, Paesmans M, Ameye L, Piccart-Gebhart MJ, de Azambuja E (2012). Role of the multidisciplinary team in breast cancer management: results from a large international survey involving 39 countries. Ann Oncol.

[3] Wright FC, De Vito C, Langer B, Hunter A (2007). Expert Panel on Multidisciplinary Cancer Conference Standards. Multidisciplinary cancer conferences: a systematic review and development of practice standards. Eur J Cancer.

[4] Patkar V, Acosta D, Davidson T, Jones A, Fox J, Keshtgar M (2011). Cancer multidisciplinary team meetings: evidence, challenges, and the role of clinical decision support technology. Int J Breast Cancer.

[5] Varga D, Wischnewsky M, Atassi Z, Wolters R, Geyer V, Strunz K, Kreienberg R, Woeckel A (2010). Does guideline-adherent therapy improve the outcome for early-onset breast cancer patients?. Oncology.

[6] Wöckel A, Kurzeder C, Geyer V, Novasphenny I, Wolters R, Wischnewsky M, Kreienberg R, Varga D (2010). Effects of guideline adherence in primary breast cancer--a 5-year multi-center cohort study of 3976 patients. Breast.

[7] Cabana MD, Rand CS, Powe NR, Wu AW, Wilson MH, Abboud PA, Rubin HR (1999). Why don’t physicians follow clinical practice guidelines? A framework for improvement. JAMA.

[8] Landercasper J, Dietrich LL, Johnson JM (2006). A breast center review of compliance with National Comprehensive Cancer Network Breast Cancer guidelines. Am J Surg.

[9] Zagouri F, Liakou P, Bartsch R, Peccatori FA, Tsigginou A, Dimitrakakis C, Zografos GC, Dimopoulos MA, Azim HA (2015). Discrepancies between ESMO and NCCN breast cancer guidelines: An appraisal. Breast.

[10] Keating NL, Landrum MB, Lamont EB, Bozeman SR, Shulman LN, McNeil BJ (2013). Tumor boards and the quality of cancer care. J Natl Cancer Inst.

[11] Hanley JA, McNeil BJ (1982). The meaning and use of the area under a receiver operating characteristic (ROC) curve. Radiology.

[12] Senkus E, Kyriakides S, Penault-Llorca F, Poortmans P, Thompson A, Zackrisson S, Cardoso F (2013). ESMO Guidelines Working Group. Primary breast cancer: ESMO Clinical Practice Guidelines for diagnosis, treatment and follow-up. Ann Oncol.

[13] Aebi S, Davidson T, Gruber G, Cardoso F, ESMO Guidelines Working Group (2011). Primary breast cancer: ESMO Clinical Practice Guidelines for diagnosis, treatment and follow-up. Ann Oncol.

[14] Aebi S, Davidson T, Gruber G, Castiglione M (2010). Primary breast cancer: ESMO Clinical Practice Guidelines for diagnosis, treatment and follow-up. Ann Oncol.

[15] Goldhirsch A, Ingle JN, Gelber RD, Coates AS, Thürlimann B, Senn HJ, Panel members (2009). Thresholds for therapies: highlights of the St Gallen International Expert Consensus on the primary therapy of early breast cancer 2009. Ann Oncol.

[16] National Comprehensive Cancer Network. Breast Cancer (Versions 2009.1, 2011.1, 2013.1, 2014.1, 2015.3). http://www.nccn.org/professionals/physician_gls/pdf/breast.pdf. Accessed 18 Sept 2015.

[17] Hall M, Frank E, Holmes G, Pfahringer B, Reutemann P, Witten IH (2009). The WEKA Data Mining Software: An Update. SIGKDD Explorations.

[18] Kesson EM, Allardice GM, George WD, Burns HJ, Morrison DS (2012). Effects of multidisciplinary team working on breast cancer survival: retrospective, comparative, interventional cohort study of 13 722 women. BMJ.

[19] Olivotto IA, Bajdik CD, Ravdin PM, Speers CH, Coldman AJ, Norris BD, Davis GJ, Chia SK, Gelmon KA (2005). Population-based validation of the prognostic model ADJUVANT! for early breast cancer. J Clin Oncol.

[20] Wishart GC, Azzato EM, Greenberg DC, Rashbass J, Kearins O, Lawrence G, Caldas C, Pharoah PD (2010). PREDICT: a new UK prognostic model that predicts survival following surgery for invasive breast cancer. Breast Cancer Res.

[21] Duric V, Stockler M (2001). Patients’ preferences for adjuvant chemotherapy in early breast cancer: a review of what makes it worthwhile. Lancet Oncol.

[22] Jansen SJ, Otten W, Stiggelbout AM (2004). Review of determinants of patients’ preferences for adjuvant therapy in cancer. J Clin Oncol.

[23] Duric VM, Stockler MR, Heritier S, Boyle F, Beith J, Sullivan A, Wilcken N, Coates AS, Simes RJ (2005). Patients’ preferences for adjuvant chemotherapy in early breast cancer: what makes AC and CMF worthwhile now?. Ann Oncol.

[24] Patkar V, Acosta D, Davidson T, Jones A, Fox J, Keshtgar M (2012). Using computerised decision support to improve compliance of cancer multidisciplinary meetings with evidence-based guidance. BMJ Open.

[25] Lamb BW, Brown KF, Nagpal K, Vincent C, Green JS, Sevdalis N (2011). Quality of care management decisions by multidisciplinary cancer teams: a systematic review. Ann Surg Oncol.

[26] Hall MA (2000). Correlation-based Feature Subset Selection for Machine Learning. Proceedings of the Seventeenth International Conference on Machine Learning.

[27] Poster Abstracts (2016). Asia-Pac J Clin Oncol.

