# Supplementary material for: Computational prediction of multidisciplinary team decision-making for adjuvant breast cancer drug therapies: a machine learning approach
Source: BMC Cancer. 2016 Dec 1;16:929. doi: 10.1186/s12885-016-2972-z (PMC5131452; doi:10.1186/s12885-016-2972-z)
Supplement: Additional file 1: — Table S1. List of clinicopathologic and outcome variables used in this analysis. Table S2. Parameters and commands used for training of the supervised learning classifiers. Table S3. List of covariates used by the trained machine learning models. Figure S1. Predictions of MDT recommendations by machine learning algorithms about adjuvant chemotherapy for each case. Figure S2. Predictions of MDT recommendations by machine learning algorithms about adjuvant endocrine therapy for each case. Figure S3. Predictions of MDT recommendations by machine learning algorithms about adjuvant trastuzumab therapy for each case. (PDF 864 kb) [file 12885_2016_2972_MOESM1_ESM.pdf]

Supplementary material

Computational prediction of multidisciplinary team decision-making for adjuvant breast cancer drug therapies: a machine learning approach

Frank PY Lin [1,2,3 *], Adrian Pokorny [1], Christina Teng [1], Rachel Dear [1,4], Richard J Epstein [1,2,3]

[1] Department of Oncology, St Vincent's Hospital, The Kinghorn Cancer Centre, Sydney, Australia
[2] Garvan Institute of Medical Research, Sydney, Australia
[3] The University of New South Wales, Sydney, NSW Australia
[4] The University of Sydney, Sydney, NSW Australia

[*]Address for correspondence:

Dr Frank Lin
Department of Oncology, The Kinghorn Cancer Centre, St Vincent's Hospital
370 Victoria St, Darlinghurst, NSW 2010, Sydney, Australia.

Tel: +612 9355 5655
Fax: +612 9355 5602
Email: f.lin@unsw.edu.au

List of supplementary tables and figures:

Table S1.	List of clinicopathologic and outcome variables used in this analysis.	2
Table S2.	Parameters and commands used for training of the supervised learning classifiers	5
Table S3.	List of covariates used by the trained machine learning models	6
Figure S1.	Predictions of MDT recommendations by machine learning algorithms about adjuvant chemotherapy for each case	9
Figure S2.	Predictions of MDT recommendations by machine learning algorithms about adjuvant endocrine therapy for each case	10
Figure S3.	Predictions of MDT recommendations by machine learning algorithms about adjuvant trastuzumab therapy for each case	11

Table S1. List of clinicopathologic and outcome variables used in this analysis.

Category	Variable	Values
**Independent (clinicopathologic) variables**	A01_MDCYear	2007, 2008, 2009, 2010, 2011, 2012, 2013, 2014, 2015
	A03_Age	*<numeric>*
	A04_Sex	Female, Male
	A05_Menopausal	Premenopausal, Perimenopausal, Postmenopausal
	A06_Status	Post biopsy, Post surgery
	A21_Prior_Biopsy	Core, FNA,Nil, Other, Core and FNA, Excisional biopsy
	A22_Prior_Surgery	"Partial mastectomy, Sentinel node biopsy", Sentinel node biopsy, "Wide local excision, Sentinel node biopsy", Axillary dissection, "Partial mastectomy, Axillary dissection", Wide local excision, "Wide local excision, Axillary dissection", Mastectomy, "Mastectomy, Sentinel node biopsy, Axillary dissection", "Partial mastectomy", "Mastectomy, Sentinel node biopsy", "Mastectomy, Axillary dissection, Chest wall excision"
	A23_Prior_Radiotherapy	Yes, No
	A24_Prior_Chemotherapy	Adjuvant, Neoadjuvant, No
	A26_Prior_HormonalTherapy	Adjuvant, Neoadjuvant, No
	A28_Prior_Herceptin	*<numeric>*
	BN20_SLN	Clear, Microscopic, Macroscopic, Isolated cells
	BN21_SLN_Involved	*<numeric>*
	BN22_SLN_Examined	*<numeric>*
	BN23_Axillary_LN_Involved	*<numeric>*
	BN24_Axillary_LN_Examined	*<numeric>*
	BN25_Extranodal_Spread	Yes, No
	BT02_Laterality	Chest wall, Right, Left, Bilateral, Axilla
	BT03_CellType	Mucinous carcinoma, Medullary carcinoma, Apocrine carcinoma, Small cell carcinoma, Myoepithelial carcinoma, Basal type, Invasive cancer NOS, Papillary carcinoma, Other malignant, Microinvasive carcinoma, Invasive lobular, Glycogen-rich clear cell, Mixed type, Tubular carcinoma, Metaplastic carcinoma, Adenoid cystic carcinoma, Micropapillary carcinoma, Invasive ductal
	BT04_Multifocal	No, Satellite lesions, Multifocal
	BT05_Size_mm	*<numeric>*
	BT06_Grade	G1, G2, G3
	BT07_LVI	Present, Probable,Absent
	BT08_PNI	Present, Absent

Category	Variable	Values
	BT09_Margin_I	Clear, <2mm, 2-5mm,>5mm,Close,Positive
	BT09_Margin_NI	Positive, <2mm, Clear, 2-5mm, >5mm
	BT10_ER	Positive, Negative, Unknown
	BT10_ER_Intensity	0 , 1+, 2+, 3+
	BT10_ER_Proportion	<*numeric*>
	BT11_PR	Positive,Negative, Unknown
	BT11_PR_Intensity	0 , 1+, 2+, 3+
	BT11_PR_Proportion	<*numeric*>
	BT12_HER2	Positive, Negative, Equivocal,Unknown
	BT12_HER2_ISH	Positive, Negative, Unknown
	BT36_BasalType	Yes, No
	BT37_CK56	Positive, Negative
	BT38_Ki67	<*numeric*>
	BX26_Second_Primary	Yes, No
	BX28_DCIS	'Low grade','High grade, Extensive','Low grade, Extensive','Extensive','Focal','High grade','Intraductal ca','Low grade, Focal','Intermediate grade','Intermediate grade, Extensive','High grade, Focal','Intermediate grade, Focal','Nil'
	BX28_DCIS_Size_mm	<*numeric*>
	BX29_LCIS	Intermediate grade, Intermediate grade, Extensive,High grade, Focal,Intermediate grade, Focal,Nil,High grade, Extensive,Low grade, Extensive,Extensive,Focal
	BX29_LCIS_Size_mm	<*numeric*>
	BX30_Benign_Lesion	Proliferative, Hyperplasia,Other,Fibroadenoma,Other, papillomatous widespread,Adenosis,Many cysts,Fibrocystic changes,Lobular hyperplasia,Ductal hyperplasia,Lobular Neoplasia & focal adenosis,IntraIntraductal papilloma
	BZ01_Subtype (derived variable)	Luminal-A-like, Luminal-B-like, HER2-overexpressed, Basal-like
	C01_Relapse_Sites	<numeric>
**Outcome variables (MDT decisions)**	RA01_Surgery	Mastectomy, Further surgery NOS,Mastectomy, Wide local excision, Further surgery NOS,Axillary dissection,Biopsy,Mastectomy, Axillary dissection,Nil,Partial mastectomy,Mastectomy, Further surgery NOS,Axillary dissection, Further surgery NOS,Wide local excision, Further surgery NOS
	RA02_Radiotherapy	Palliative, Other, Not recommended, Recommended,For discussion, Recommended if WLE/BCS
	RA02_Radiotherapy_aggressive	Recommended, Not recommended
	RA02_Radiotherapy_conservative	Recommended, Not recommended
	RA02_Radiotherapy_tutti	Recommended, Not recommended, For discussion
	RA03_Chemotherapy	Adjuvant, For discussion, Neoadjuvant, Palliative, Not recommended
	RA03_Chemotherapy_aggressive	Recommended, Not recommended

Category	Variable	Values
	RA03_Chemotherapy_conservative	Recommended, Not recommended
	RA03_Chemotherapy_tutti	Recommended, Not recommended, For discussion
	RA04_Endocrine	For discussion, Adjuvant,Not recommended,Palliative
	RA04_Endocrine_aggressive	Recommended, Not recommended
	RA04_Endocrine_conservative	Recommended, Not recommended
	RA04_Endocrine_tutti	Recommended, Not recommended, For discussion
	RA05_Herceptin	Not recommended, Adjuvant, For discussion
	RA05_Herceptin_aggressive	Recommended, Not recommended
	RA05_Herceptin_conservative	Recommended, Not recommended
	RA05_Herceptin_tutti	Recommended, Not recommended, For discussion
	RA08_Genetics	Not required, Recommended, For discussion
	RA09_Psychologist	Not required, Recommended, For discussion
	RA13_Dissenters	Yes, No
	RA14_DissentReason	'Chemotherapy','Chemotherapy, Oncotype','Chemotherapy, Herceptin','Oncotype','Chemotherapy, Further investigation','Genetics','Endocrine','Further investigation','Chemotherapy, Radiotherapy','Herceptin','Radiotherapy','Surgery','Endocrine, Radiotherapy','Chemotherapy, Endocrine'
**Outcome variables**	RG01_Chemotherapy_ESMO_tutti	Recommended, Not recommended, For discussion
	RG02_Endocrine_ESMO_tutti	Recommended, Not recommended
	RG03_Herceptin_ESMO_tutti	Recommended, Not recommended
	RG01_Chemotherapy_ESMO_aggressive	Recommended, Not recommended
	RG02_Endocrine_ESMO_aggressive	Recommended, Not recommended
	RG03_Herceptin_ESMO_aggressive	Recommended, Not recommended
	RG01_Chemotherapy_ESMO_conservative	Recommended, Not recommended
	RG02_Endocrine_ESMO_conservative	Recommended, Not recommended
	RG03_Herceptin_ESMO_conservative	Recommended, Not recommended
	RG01_Chemotherapy_NCCN_tutti	Recommended, Not recommended, For discussion
	RG02_Endocrine_NCCN_tutti	Recommended, Not recommended, For discussion
	RG03_Herceptin_NCCN_tutti	Recommended, Not recommended, For discussion
	RG01_Chemotherapy_NCCN_aggressive	Recommended, Not recommended
	RG02_Endocrine_NCCN_aggressive	Recommended, Not recommended
	RG03_Herceptin_NCCN_aggressive	Recommended, Not recommended
	RG01_Chemotherapy_NCCN_conservative	Recommended, Not recommended
	RG02_Endocrine_NCCN_conservative	Recommended, Not recommended
	RG03_Herceptin_NCCN_conservative	Recommended, Not recommended

Table S2. Parameters and commands used for training of the supervised learning classifiers

Algorithm	Parameter selection	WEKA command
Naive Bayes	None	weka.classifiers.bayes.NaiveBayes
Logistic regression	Ridge value = 10^-8	weka.classifiers.functions.Logistic -R 1.0E-8 -M -1
J48 decision tree	Minimum confidence of 0.25 with minimum of 2 objects in a branch	weka.classifiers.trees.J48 -C 0.25 -M 2
Multiclass alternating decision tree (ADTree)	Ten boosting iterations	weka.classifiers.trees.LADTree -B 10
Decision table	Best-first selection and Leave-one-out CV	weka.classifiers.rules.DecisionTable -X 1 -R -S "weka.attributeSelection.BestFirst"
Support vector machine (SVM) with Polynomial kernel	Fit logistic models to SVM outputs.<br>No internal cross-validation.	weka.classifiers.functions.SMO -M -C 1.0 -L 0.0010 -P 1.0E-12 -N 0 -V -1 -W 1 -K weka.classifiers.functions.supportVector.PolyKernel
SVM with radial basis function (RBF) kernel	Fit logistic models to SVM outputs.<br>No internal cross-validation.	weka.classifiers.functions.SMO -M -C 1.0 -L 0.0010 -P 1.0E-12 -N 0 -V -1 -W 1 -K weka.classifiers.functions.supportVector.RBFKernel
Nearest neighbour classifier	hold-one-out evaluation on the training data	weka.classifiers.lazy.IBk -X -K 5 -I
OneR classifier	Minimum number of objects in a bucket = 3	weka.classifiers.rules.OneR -B 3
Ripple-down rules	3-fold IREP, and default weight within a split	weka.classifiers.rules.Ridor -F 3 -S 1 -N 2.0
Naive Bayes (bagged)	Bagging with 100% bag size and 10 iterations	weka.classifiers.meta.Bagging -P 100 -S 1 -I 10 -W weka.classifiers.bayes.NaiveBayes
J48 decision tree (bagged)	Bagging with 100% bag size and 10 iterations	weka.classifiers.meta.Bagging -P 100 -S 1 -I 10 -W weka.classifiers.trees.J48 -- -C 0.25 -M 2
Multiclass ADTree (bagged)	Bagging with 100% bag size and 10 iterations	weka.classifiers.meta.Bagging -P 100 -S 1 -I 10 -W weka.classifiers.trees.LADTree -- -B 10
OneR (bagged)	Bagging with 100% bag size and 10 iterations	weka.classifiers.meta.Bagging -P 100 -S 1 -I 10 -W weka.classifiers.rules.OneR -- -B 3
Ridor (bagged)	Bagging with 100% bag size and 10 iterations	weka.classifiers.meta.Bagging -P 100 -S 1 -I 10 -W weka.classifiers.rules.Ridor -- -F 3 -S 1 -N 2.0
SVM with Polynomial kernel (bagged)	Bagging with 100% bag size and 10 iterations	weka.classifiers.meta.Bagging -P 100 -S 1 -I 10 -W weka.classifiers.functions.SMO -- -M -C 1.0 -L 0.0010 -P 1.0E-12 -N 0 -V -1 -W 1 -K weka.classifiers.functions.supportVector.PolyKernel
SVM with RBF kernel (bagged)	Bagging with 100% bag size and 10 iterations	weka.classifiers.meta.Bagging -P 100 -S 1 -I 10 -W weka.classifiers.functions.SMO -- -M -C 1.0 -L 0.0010 -P 1.0E-12 -N 0 -V -1 -W 1 -K weka.classifiers.functions.supportVector.RBFKernel
Logistic regression (bagged)	Bagging with 100% bag size and 10 iterations	weka.classifiers.meta.Bagging -P 100 -S 1 -I 10 -W weka.classifiers.functions.Logistic -- -R 1.0E-8 -M -1

Table S3. List of covariates used by the trained machine learning models

Algorithm	Adjuvant chemotherapy		Adjuvant endocrine therapy		Adjuvant trastuzumab	
	No ensemble learning	Bootstrap-aggregated	No ensemble learning	Bootstrap-aggregated	No ensemble learning	Bootstrap-aggregated
OneR classifier	BT06_Grade	A03_Age<br>BT06_Grade	BT10_ER	BT10_ER<br>BZ01_Subtype	BT12_HER2_ISH	BT12_HER2_ISH
Decision table	A03_Age<br>A04_Sex<br>BN23_Axillary_LN_Involved<br>BT06_Grade	Not evaluated	BT10_ER<br>BT10_ER_Proportion	Not evaluated	A24_Prior_Chemotherapy<br>BT06_Grade<br>BT09_Margin_NI<br>BT12_HER2_ISH	Not evaluated
J48 decision tree	A03_Age<br>BN21_SLN_Involved<br>BN22_SLN_Examined<br>BN23_Axillary_LN_Involved<br>BN24_Axillary_LN_Examined<br>BN25_Extranodal_Spread<br>BT05_Size_mm<br>BT06_Grade<br>BT07_LVI<br>BT09_Margin_I<br>BT10_ER<br>BT12_HER2<br>BT12_HER2_ISH<br>BT38_Ki67	A03_Age<br>A04_Sex<br>A05_Menopausal<br>A06_Status<br>A21_Prior_Biopsy<br>A22_Prior_Surgery<br>BN20_SLN<br>BN21_SLN_Involved<br>BN22_SLN_Examined<br>BN23_Axillary_LN_Involved<br>BN24_Axillary_LN_Examined<br>BN25_Extranodal_Spread<br>BT03_CellType<br>BT05_Size_mm<br>BT06_Grade<br>BT07_LVI<br>BT09_Margin_I<br>BT09_Margin_NI<br>BT10_ER<br>BT10_ER_Proportion<br>BT11_PR<br>BT12_HER2<br>BT12_HER2_ISH<br>BT38_Ki67<br>BX28_DCIS<br>BX28_DCIS_Size_mm<br>BX29_LCIS<br>BZ01_Subtype	A03_Age<br>BN20_SLN<br>BN23_Axillary_LN_Involved<br>BT05_Size_mm<br>BT06_Grade<br>BT10_ER<br>BT11_PR	A03_Age<br>A04_Sex<br>A05_Menopausal<br>A21_Prior_Biopsy<br>A22_Prior_Surgery<br>BN20_SLN<br>BN21_SLN_Involved<br>BN22_SLN_Examined<br>BN23_Axillary_LN_Involved<br>BN24_Axillary_LN_Examined<br>BT03_CellType<br>BT05_Size_mm<br>BT06_Grade<br>BT09_Margin_I<br>BT10_ER<br>BT11_PR<br>BT12_HER2<br>BT12_HER2_ISH<br>BT37_CK56<br>BX28_DCIS<br>BX28_DCIS_Size_mm<br>BZ01_Subtype	A03_Age<br>BT05_Size_mm<br>BT12_HER2<br>BT12_HER2_ISH	A03_Age<br>BN21_SLN_Involved<br>BN22_SLN_Examined<br>BN24_Axillary_LN_Examined<br>BT03_CellType<br>BT05_Size_mm<br>BT06_Grade<br>BT10_ER<br>BT11_PR<br>BT12_HER2<br>BT12_HER2_ISH<br>BX29_LCIS<br>BZ01_Subtype

Algorithm	Adjuvant chemotherapy		Adjuvant endocrine therapy		Adjuvant trastuzumab	
	**No ensemble learning**	**Bootstrap-aggregated**	**No ensemble learning**	**Bootstrap-aggregated**	**No ensemble learning**	**Bootstrap-aggregated**
Multiclass ADTree	A03_Age<br>BN20_SLN<br>BT05_Size_mm<br>BT06_Grade<br>BT07_LVI<br>BT10_ER<br>BT12_HER2	A03_Age<br>BN20_SLN<br>BT05_Size_mm<br>BT06_Grade<br>BT07_LVI<br>BT09_Margin_I<br>BT09_Margin_NI<br>BT10_ER<br>BT12_HER2<br>BT37_CK56<br>BZ01_Subtype	A03_Age<br>BN20_SLN<br>BT05_Size_mm<br>BT06_Grade<br>BT07_LVI<br>BT10_ER<br>BT11_PR	A03_Age<br>A05_Menopausal<br>A22_Prior_Surgery<br>BN20_SLN<br>BT03_CellType<br>BT05_Size_mm<br>BT06_Grade<br>BT07_LVI<br>BT08_PNI<br>BT09_Margin_I<br>BT09_Margin_NI<br>BT10_ER<br>BT10_ER_Intensity<br>BT11_PR<br>BT11_PR_Intensity<br>BT12_HER2<br>BT12_HER2_ISH<br>BT36_BasalType<br>BT37_CK56<br>BX28_DCIS<br>BX30_Benign_Lesion	A03_Age<br>BT02_Laterality<br>BT03_CellType<br>BT05_Size_mm<br>BT06_Grade<br>BT12_HER2<br>BT12_HER2_ISH	A03_Age<br>A05_Menopausal<br>A22_Prior_Surgery<br>BN20_SLN<br>BN23_Axillary_LN_Involved<br>BT02_Laterality<br>BT03_CellType<br>BT05_Size_mm<br>BT06_Grade<br>BT08_PNI<br>BT09_Margin_NI<br>BT10_ER_Intensity<br>BT12_HER2<br>BT12_HER2_ISH<br>BT38_Ki67<br>BX28_DCIS<br>BZ01_Subtype
Ripple-down rules	A03_Age<br>BN20_SLN<br>BN21_SLN_Involved<br>BN22_SLN_Examined<br>BN23_Axillary_LN_Involved<br>BN24_Axillary_LN_Examined<br>BN25_Extranodal_Spread<br>BT05_Size_mm<br>BT06_Grade<br>BT07_LVI<br>BT09_Margin_I<br>BT10_ER<br>BT12_HER2<br>BT12_HER2_ISH<br>BT38_Ki67<br>BX29_LCIS<br>BZ01_Subtype	A03_Age<br>A06_Status<br>A22_Prior_Surgery<br>BN20_SLN<br>BN21_SLN_Involved<br>BN22_SLN_Examined<br>BN23_Axillary_LN_Involved<br>BN24_Axillary_LN_Examined<br>BN25_Extranodal_Spread<br>BT03_CellType<br>BT04_Multifocal<br>BT05_Size_mm<br>BT06_Grade<br>BT07_LVI<br>BT09_Margin_I<br>BT09_Margin_NI<br>BT10_ER<br>BT10_ER_Proportion<br>BT11_PR<br>BT11_PR_Intensity	A03_Age<br>A22_Prior_Surgery<br>BN22_SLN_Examined<br>BN23_Axillary_LN_Involved<br>BN24_Axillary_LN_Examined<br>BT05_Size_mm<br>BT06_Grade<br>BT10_ER<br>BT11_PR<br>BT12_HER2<br>BT12_HER2_ISH<br>BT38_Ki67<br>BX28_DCIS_Size_mm	A03_Age<br>A05_Menopausal<br>A06_Status<br>A21_Prior_Biopsy<br>A22_Prior_Surgery<br>A24_Prior_Chemotherapy<br>BN20_SLN<br>BN21_SLN_Involved<br>BN22_SLN_Examined<br>BN23_Axillary_LN_Involved<br>BN24_Axillary_LN_Examined<br>BN25_Extranodal_Spread<br>BT02_Laterality<br>BT03_CellType<br>BT05_Size_mm<br>BT06_Grade<br>BT07_LVI<br>BT09_Margin_I<br>BT10_ER<br>BT10_ER_Proportion	A03_Age<br>BN24_Axillary_LN_Examined<br>BT05_Size_mm<br>BT06_Grade<br>BT11_PR<br>BT11_PR_Proportion<br>BT12_HER2<br>BT12_HER2_ISH	A03_Age<br>A21_Prior_Biopsy<br>A22_Prior_Surgery<br>BN21_SLN_Involved<br>BN22_SLN_Examined<br>BN23_Axillary_LN_Involved<br>BN24_Axillary_LN_Examined<br>BT03_CellType<br>BT05_Size_mm<br>BT06_Grade<br>BT10_ER_Intensity<br>BT11_PR<br>BT12_HER2<br>BT12_HER2_ISH<br>BT38_Ki67<br>BX28_DCIS<br>BZ01_Subtype

Algorithm	Adjuvant chemotherapy		Adjuvant endocrine therapy		Adjuvant trastuzumab	
	**No ensemble learning**	**Bootstrap-aggregated**	**No ensemble learning**	**Bootstrap-aggregated**	**No ensemble learning**	**Bootstrap-aggregated**
Ripple-down rules (Continued)		BT11_PR_Proportion<br>BT12_HER2<br>BT12_HER2_ISH<br>BT38_Ki67<br>BX28_DCIS<br>BX28_DCIS_Size_mm<br>BX29_LCIS<br>BZ01_Subtype		BT11_PR<br>BT11_PR_Intensity<br>BT11_PR_Proportion<br>BT12_HER2<br>BT12_HER2_ISH<br>BT38_Ki67<br>BX28_DCIS<br>BX28_DCIS_Size_mm<br>BX29_LCIS<br>BX30_Benign_Lesion<br>BZ01_Subtype		

All variables are included as covariates of model in Naive Bayes, Logistic regression, support vector machine, nearest neighhour classifiers, as well as the bootstrap-aggregated counterparts.

Supplementary Figure S1. Predictions of MDT recommendations by machine learning algorithms about adjuvant chemotherapy for each case

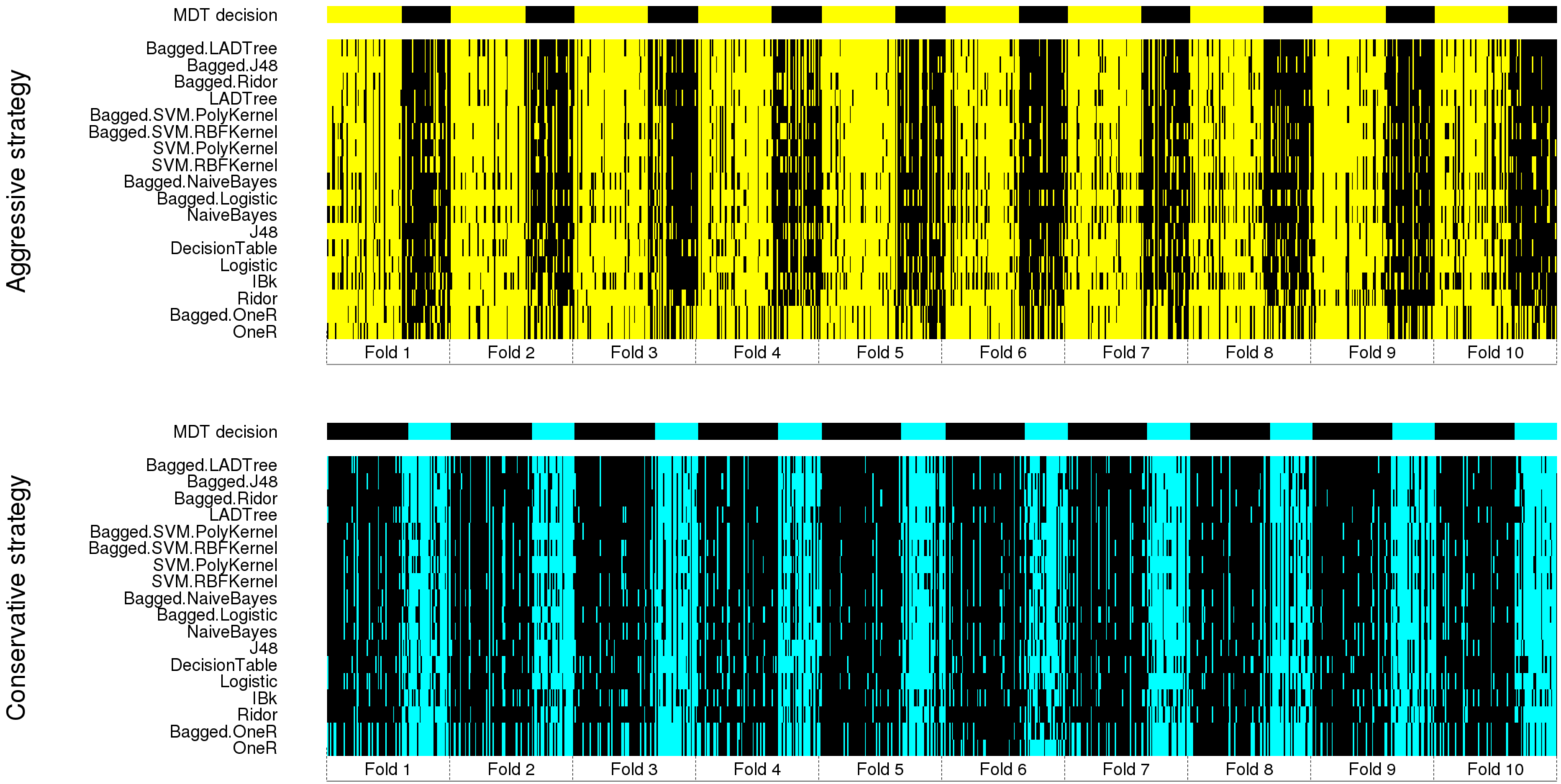

Note: Stratified 10-fold cross-validation. Coloured (yellow or cyan): Recommended; Black: Not recommended

Supplementary Figure S2. Predictions of MDT recommendations by machine learning algorithms about adjuvant endocrine therapy for each case

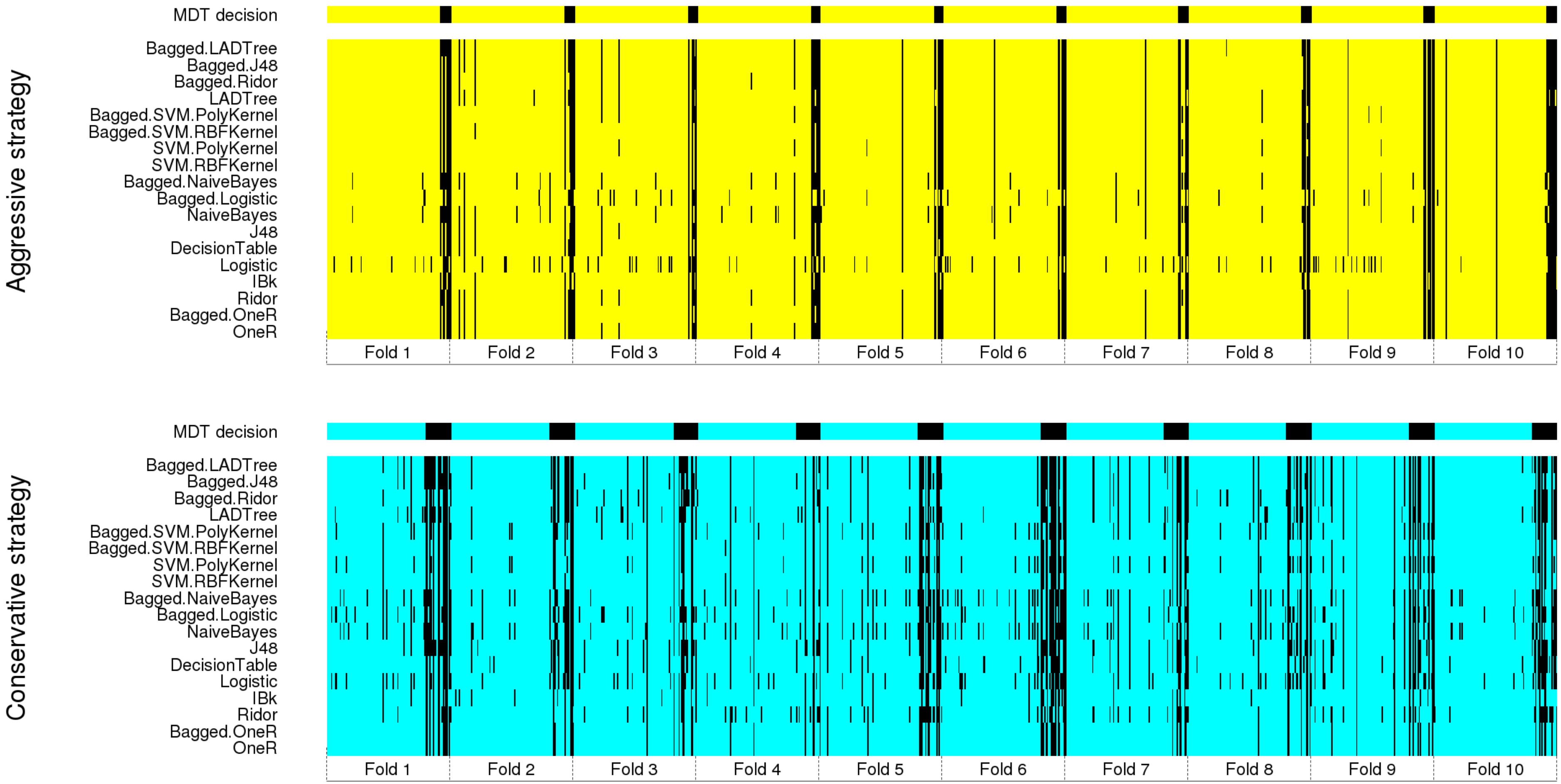

Note: Stratified 10-fold cross-validation. Coloured (yellow or cyan): Recommended; Black: Not recommended

Supplementary Figure S3. Predictions of MDT recommendations by machine learning algorithms about adjuvant trastuzumab therapy for each case

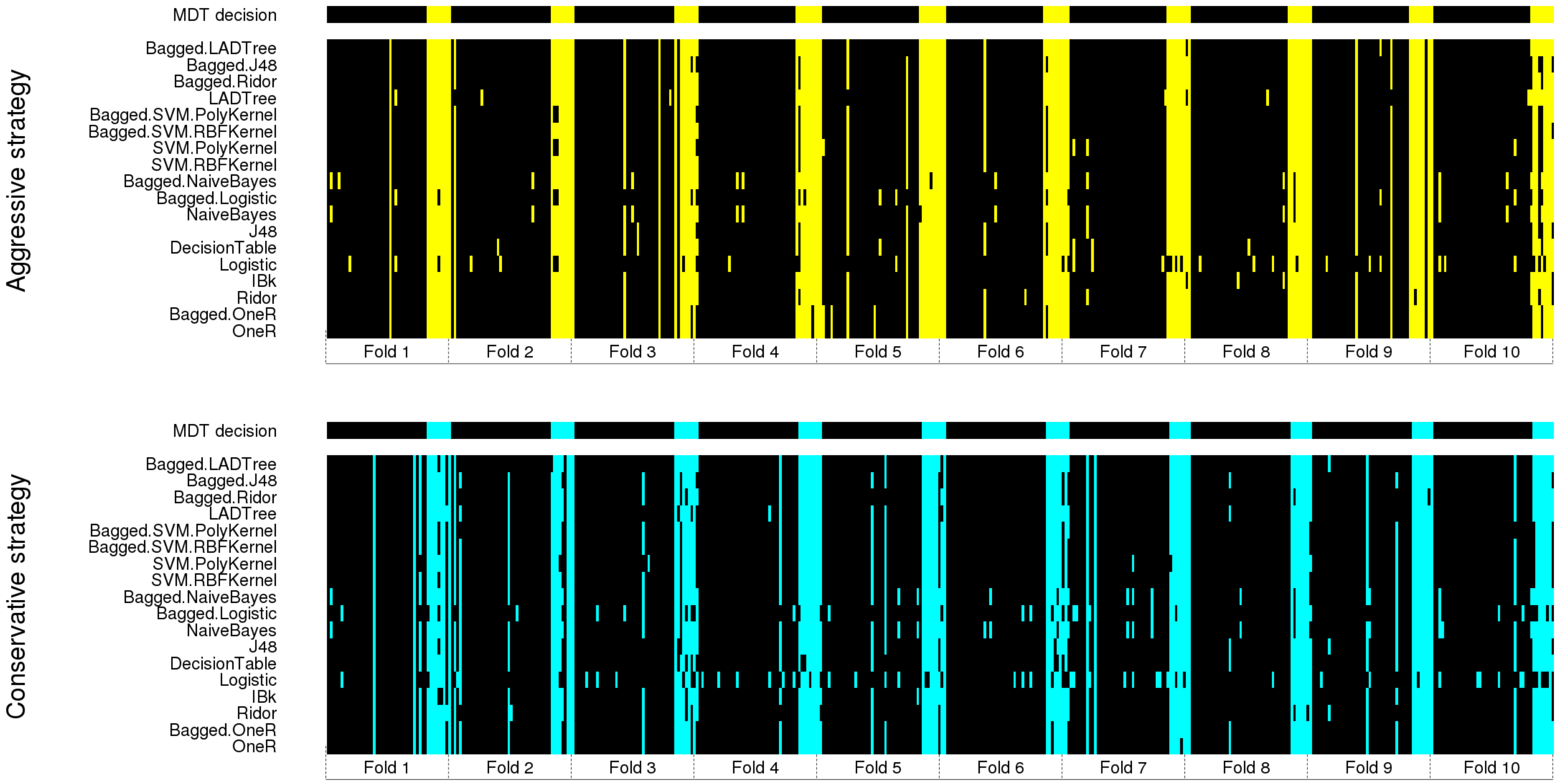

Note: Stratified 10-fold cross-validation. Coloured (yellow or cyan): Recommended; Black: Not recommended
